# Supplementary material for: Single-nucleus multiomics reveals the gene-regulatory networks underlying sex determination of murine primordial germ cells
Source: bioRxiv. 2024 Sep 25:2024.02.19.581036. Preprint. [Version 3] doi: 10.1101/2024.02.19.581036 (PMC11463670; doi:10.1101/2024.02.19.581036)
Supplement: Supplement 2 [file NIHPP2024.02.19.581036v3-supplement-2.pdf]

bioRxiv preprint doi: https://doi.org/10.1101/2024.02.19.581036; this version posted September 25, 2024. The copyright holder for this preprint (which was not certified by peer review) is the author/funder. This article is a US Government work. It is not subject to copyright under 17 USC 105 and is also made available for use under a CC0 license.

Supplementary Table 1. Sequencing and QC statistics for single-nucleus multiome libraries of E11.5-E13.5 XX and XY gonads.

Sample	Pooled Paired Gonads (Number)	Raw Reads	Barcode	Mapped	Saturation	Cells	Reads/cell	Median UMI/cell	Median genes/cell
**E11.5 F_R1**	19	448,115,792	93%	91%	38%	20,000	22,406	3,665	2,030
**E11.5 F_R2**	11	812,813,546	94%	92%	68%	10,008	81,216	8,184	2,798
**E11.5 M_R1**	18	193,672,880	93%	94%	35%	8,080	23,969	2,790	1,708
**E11.5 M_R2**	12	719,219,983	94%	91%	64%	4,960	145,326	5,934	3,512
**E12.5 F_R1**	12	498,266,895	93%	94%	43%	18,696	26,651	4,620	2,422
**E12.5 F_R2**	11	654,449,771	94%	91%	66%	4,949	131,946	8,351	3,504
**E12.5 M_R1**	8	425,422,729	93%	91%	44%	14,897	28,558	5,268	2,682
**E12.5 M_R2**	10	951,070,062	94%	92%	74%	5,864	162,188	9,194	3,446

bioRxiv preprint doi: https://doi.org/10.1101/2024.02.19.581036; this version posted September 25, 2024. The copyright holder for this preprint (which was not certified by peer review) is the author/funder. This article is a US Government work. It is not subject to copyright under 17 USC 105 and is also made available for use under a CC0 license.

**E13.5 F_R1**	3	135,990,358	95%	92%	86%	1,555	83,036	3,608	2,121
**E13.5 F_R2**	5	398,709,188	95%	92%	73%	6,369	74,137	6,013	2,831
**E13.5 M_R1**	3	129,120,442	94%	94%	82%	2,040	66,662	5,132	2,642
**E13.5 M_R2**	7	472,180,945	94%	91%	71%	6,597	60,438	6,546	2,969
